# 4,6-Dichloro-5-(2-meth­oxy­phen­oxy)-2,2′-bipyrimidine

**DOI:** 10.1107/S160053681101419X

**Published:** 2011-05-07

**Authors:** Tian-tian Ren, Zhong Zhang, Cong-cong Zhong, Zhong-zhi Yang, Zhan-wang Shi

**Affiliations:** a Department of Chemistry, Guangxi University for Nationalities, Nanning 530006, People’s Republic of China

## Abstract

In the title compound, C_15_H_10_Cl_2_N_4_O_2_, the dichloro­pyrimidine and meth­oxy­phen­oxy parts are approximately perpendicular [dihedral angle = 89.9 (9)°]. The dihedral angle between the two pyrimidine rings is 36.3 (4)° In the crystal, there are no hydrogen bonds but the mol­ecules are held together by short inter­molecular C⋯N [3.206 (3) Å] contacts and C—H⋯π inter­actions.

## Related literature

For the use of 2,2′-bipyrimidine as a ligand in inorganic and organometallic chemistry, see: Fabrice *et al.* (2008 ▶); Hunziker & Ludi (1977 ▶). It was first synthesized by Bly and Mellon (1962 ▶) utilizing the Ullmann coupling of 2-bromo­pyrimidine in the presence of metallic copper.
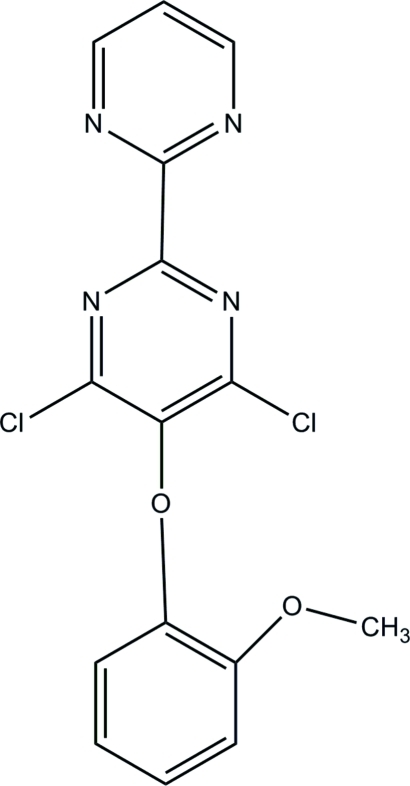


## Experimental

### 

#### Crystal data

C_15_H_10_Cl_2_N_4_O_2_
*M* *_r_* = 349.17
Monoclinic, 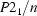

*a* = 10.716 (2) Å
*b* = 8.1112 (18) Å
*c* = 18.601 (5) Å
β = 106.486 (3)°
*V* = 1550.3 (6) Å^3^
*Z* = 4
Mo *K*α radiation
μ = 0.43 mm^−1^
*T* = 296 K
0.22 × 0.20 × 0.18 mm

#### Data collection

Siemens SMART CCD area-detector diffractometer
Absorption correction: multi-scan (*SADABS*; Sheldrick, 1996 ▶) *T*_min_ = 0.911, *T*_max_ = 0.926
8106 measured reflections
2733 independent reflections
2319 reflections with *I* > 2σ(*I*)
*R*_int_ = 0.027

#### Refinement

*R*[*F* ^2^ > 2σ(*F* ^2^)] = 0.042
*wR*(*F* ^2^) = 0.114
*S* = 1.08
2733 reflections
209 parameters
H-atom parameters constrained
Δρ_max_ = 0.26 e Å^−3^
Δρ_min_ = −0.54 e Å^−3^

### 

Data collection: *SMART* (Siemens, 1996 ▶); cell refinement: *SAINT* (Siemens, 1996 ▶); data reduction: *SAINT*; program(s) used to solve structure: *SHELXS97* (Sheldrick, 2008 ▶); program(s) used to refine structure: *SHELXL97* (Sheldrick, 2008 ▶); molecular graphics: *SHELXTL* (Sheldrick, 2008 ▶); software used to prepare material for publication: *SHELXTL*.

## Supplementary Material

Crystal structure: contains datablocks I, global. DOI: 10.1107/S160053681101419X/fl2331sup1.cif

Structure factors: contains datablocks I. DOI: 10.1107/S160053681101419X/fl2331Isup2.hkl

Additional supplementary materials:  crystallographic information; 3D view; checkCIF report

## Figures and Tables

**Table 1:** C—H⋯π interactions (Å, °) *Cg* is the centroid of the C9–C14 ring.

*D*—H⋯*A*	*D*—H	H⋯*A*	*D*⋯*A*	*D*—H⋯*A*
C1—H1⋯*Cg*^i^	0.93	2.87	3.760 (3)	161

Symmetry code: (i) 
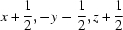
.

**Fig. 1.**
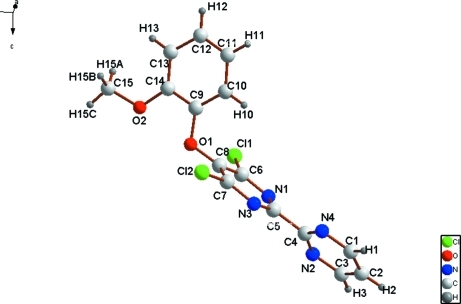
The structure of the title compound (I) showing 50% probability displacement ellipsoids and the atom-numbering scheme.

**Fig. 2.**
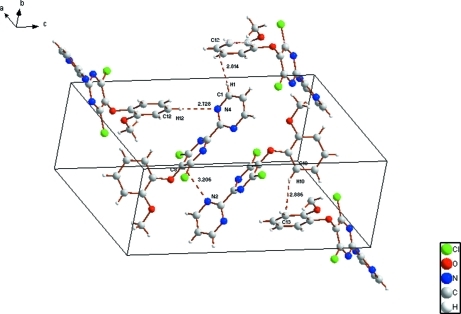
Crystal packing of (I) showing the short contacts interactions as dashed lines.

## supplementary crystallographic information

### Comment

2,2'-Bipyrimidine has been used as a ligand in inorganic and organometallic chemistry (Hunziker & Ludi, 1977; Fabrice *et al.*, 2008) and exhibits remarkable stability in acidic medium. It was first synthesized by Bly and Mellon (1962) utilizing the Ullmann coupling of 2-bromopyrimidine in the presence of metallic copper. As part of our studies on the synthesis and characterization of these compounds, we report here the crystal structure of (I).

In (I) the dichloropyrimidine and the methoxyphenoxy are approximately perpendicular (dihedral angle of 89.90 (91) °). The dihedral angle between the two pyrimidine rings is 36.25 (38)° (Fig. 1).

The molecules are linked by intermolecular C—H···N , C—H···C and C···N short contacts. C12—H12···N4, C12···N4=3.639 (3), H12···N4=2.728 (3), and C12—H12···N4=166.49; C8···N2=3.206 (3); C1—H1···C12, C1···C12=3.725 (3), H1···C12=2.814 (3), and C1—H1···C12=166.14; C10—H10···C13, C10···C13=3.694 (3)), H10···C13=2.886 (3), and C13—H10···C10=146.05.

### Experimental

A solution of 4,6-Dichloro-5-(2-methoxyphenoxy)-2,2'-bipyrimidine (10 mmol) in 50ml toluene was refluxed for 1h with stirring then filtered, washed several times with ethanol and dried *in vacuo*, to produce a powder of the title compound. The powder was added to 50ml toluene with stirring until completely dissolved. Finally, colourless crystals suitable for data collection were obtained by slow evaporation of the toluene at room temperature. Elemental analysis calculate: Elemental analysis: found: C, 51.60; H, 2.89; N, 16.05; calc. for C_15_H_10_Cl_2_N_4_O_2_: C,51.53; H, 2.93; N, 16.11.

### Refinement

Data collection-2102 independent reflections but 2101 in Refinement. H atoms on C atoms were positoned geometrically and refined using a riding model with C—H = 0.96Å and *U*_iso_(H) = 1.2*U*_eq_(C).

### Crystal data

**Table:**

C_15_H_10_Cl_2_N_4_O_2_	*F*(000) = 712
*M**_r_* = 349.17	*D*_x_ = 1.496 Mg m^−^^3^
Monoclinic, *P*2_1_/*n*	Mo *K*α radiation, λ = 0.71073 Å
*a* = 10.716 (2) Å	Cell parameters from 3720 reflections
*b* = 8.1112 (18) Å	θ = 2.6–27.4°
*c* = 18.601 (5) Å	µ = 0.43 mm^−^^1^
β = 106.486 (3)°	*T* = 296 K
*V* = 1550.3 (6) Å^3^	Block, colourless
*Z* = 4	0.22 × 0.20 × 0.18 mm

### Data collection

**Table:**

Bruker SMART CCD area-detector diffractometer	2733 independent reflections
Radiation source: fine-focus sealed tube	2319 reflections with *I* > 2σ(*I*)
graphite	*R*_int_ = 0.027
φ and ω scans	θ_max_ = 25.0°, θ_min_ = 2.0°
Absorption correction: multi-scan (*SADABS*; Sheldrick, 1996)	*h* = −12→12
*T*_min_ = 0.911, *T*_max_ = 0.926	*k* = −9→9
8106 measured reflections	*l* = −22→16

### Refinement

**Table:**

Refinement on *F*^2^	Primary atom site location: structure-invariant direct methods
Least-squares matrix: full	Secondary atom site location: difference Fourier map
*R*[*F*^2^ > 2σ(*F*^2^)] = 0.042	Hydrogen site location: inferred from neighbouring sites
*wR*(*F*^2^) = 0.114	H-atom parameters constrained
*S* = 1.08	*w* = 1/[σ^2^(*F*_o_^2^) + (0.0522*P*)^2^ + 0.7172*P*] where *P* = (*F*_o_^2^ + 2*F*_c_^2^)/3
2733 reflections	(Δ/σ)_max_ = 0.001
209 parameters	Δρ_max_ = 0.26 e Å^−^^3^
0 restraints	Δρ_min_ = −0.54 e Å^−^^3^

### Special details

**Table:**

Geometry. All esds (except the esd in the dihedral angle between two l.s. planes) are estimated using the full covariance matrix. The cell esds are taken into account individually in the estimation of esds in distances, angles and torsion angles; correlations between esds in cell parameters are only used when they are defined by crystal symmetry. An approximate (isotropic) treatment of cell esds is used for estimating esds involving l.s. planes.
Refinement. Refinement of F^2^ against ALL reflections. The weighted R-factor wR and goodness of fit S are based on F^2^, conventional R-factors R are based on F, with F set to zero for negative F^2^. The threshold expression of F^2^ > 2sigma(F^2^) is used only for calculating R-factors(gt) etc. and is not relevant to the choice of reflections for refinement. R-factors based on F^2^ are statistically about twice as large as those based on F, and R- factors based on ALL data will be even larger.

### Fractional atomic coordinates and isotropic or equivalent isotropic displacement parameters (Å^2^)

**Table:**

	*x*	*y*	*z*	*U*_iso_*/*U*_eq_	
Cl2	0.35488 (7)	0.23438 (8)	0.95225 (4)	0.0698 (2)	
Cl1	−0.12322 (6)	0.00465 (9)	0.81525 (4)	0.0697 (2)	
O1	0.09431 (17)	0.24803 (18)	0.84012 (8)	0.0514 (4)	
N1	0.04052 (18)	−0.1321 (2)	0.93028 (10)	0.0461 (4)	
O2	−0.01418 (17)	0.4733 (2)	0.74474 (9)	0.0564 (4)	
N3	0.25212 (18)	−0.0303 (2)	0.99122 (9)	0.0434 (4)	
N2	0.07696 (19)	−0.3406 (2)	1.05310 (10)	0.0509 (5)	
C4	0.1789 (2)	−0.2855 (3)	1.03313 (11)	0.0394 (5)	
C5	0.1558 (2)	−0.1388 (3)	0.98205 (11)	0.0399 (5)	
N4	0.29885 (19)	−0.3461 (2)	1.05286 (11)	0.0504 (5)	
C9	0.1043 (2)	0.2281 (3)	0.76750 (11)	0.0401 (5)	
C8	0.1164 (2)	0.1136 (3)	0.88636 (11)	0.0437 (5)	
C13	0.0568 (2)	0.3436 (3)	0.64467 (12)	0.0480 (5)	
H13	0.0179	0.4242	0.6099	0.058*	
C14	0.0468 (2)	0.3523 (3)	0.71727 (11)	0.0403 (5)	
C12	0.1243 (2)	0.2156 (3)	0.62364 (12)	0.0512 (6)	
H12	0.1311	0.2112	0.5749	0.061*	
C6	0.2308 (2)	0.0942 (3)	0.94339 (11)	0.0446 (5)	
C2	0.2172 (3)	−0.5496 (3)	1.11929 (13)	0.0580 (6)	
H2	0.2305	−0.6438	1.1490	0.070*	
C15	−0.0513 (3)	0.6175 (3)	0.69994 (17)	0.0712 (8)	
H15A	−0.1114	0.5880	0.6528	0.107*	
H15B	0.0245	0.6674	0.6914	0.107*	
H15C	−0.0920	0.6943	0.7255	0.107*	
C10	0.1703 (2)	0.1008 (3)	0.74643 (12)	0.0501 (6)	
H10	0.2076	0.0185	0.7806	0.060*	
C11	0.1813 (2)	0.0952 (3)	0.67400 (13)	0.0537 (6)	
H11	0.2272	0.0100	0.6596	0.064*	
C3	0.0986 (3)	−0.4745 (3)	1.09680 (13)	0.0564 (6)	
H3	0.0303	−0.5180	1.1124	0.068*	
C7	0.0238 (2)	−0.0064 (3)	0.88345 (12)	0.0457 (5)	
C1	0.3157 (3)	−0.4805 (3)	1.09624 (14)	0.0589 (7)	
H1	0.3978	−0.5289	1.1113	0.071*	

### Atomic displacement parameters (Å^2^)

**Table:**

	*U*^11^	*U*^22^	*U*^33^	*U*^12^	*U*^13^	*U*^23^
Cl2	0.0869 (5)	0.0571 (4)	0.0554 (4)	−0.0231 (3)	0.0039 (3)	0.0114 (3)
Cl1	0.0610 (4)	0.0732 (5)	0.0600 (4)	0.0094 (3)	−0.0073 (3)	0.0149 (3)
O1	0.0831 (11)	0.0397 (9)	0.0335 (8)	0.0168 (8)	0.0197 (8)	0.0094 (6)
N1	0.0521 (11)	0.0438 (10)	0.0403 (10)	0.0047 (8)	0.0095 (8)	0.0056 (8)
O2	0.0784 (11)	0.0468 (9)	0.0461 (9)	0.0198 (8)	0.0209 (8)	0.0144 (7)
N3	0.0577 (11)	0.0390 (10)	0.0316 (9)	0.0020 (8)	0.0094 (8)	0.0035 (7)
N2	0.0617 (12)	0.0495 (11)	0.0446 (10)	0.0045 (9)	0.0203 (9)	0.0101 (9)
C4	0.0534 (12)	0.0357 (11)	0.0291 (10)	0.0028 (9)	0.0115 (9)	−0.0003 (8)
C5	0.0518 (12)	0.0379 (11)	0.0307 (10)	0.0062 (9)	0.0127 (9)	0.0015 (8)
N4	0.0558 (11)	0.0447 (11)	0.0490 (11)	0.0080 (9)	0.0121 (9)	0.0089 (9)
C9	0.0471 (11)	0.0431 (12)	0.0286 (10)	−0.0003 (9)	0.0084 (8)	0.0024 (8)
C8	0.0639 (14)	0.0369 (11)	0.0313 (10)	0.0119 (10)	0.0151 (10)	0.0058 (9)
C13	0.0564 (13)	0.0493 (13)	0.0362 (11)	−0.0062 (10)	0.0095 (10)	0.0082 (10)
C14	0.0436 (11)	0.0402 (12)	0.0357 (11)	−0.0011 (9)	0.0087 (9)	0.0036 (9)
C12	0.0572 (14)	0.0628 (15)	0.0355 (12)	−0.0119 (11)	0.0164 (10)	−0.0054 (11)
C6	0.0624 (13)	0.0374 (11)	0.0334 (10)	−0.0003 (10)	0.0124 (10)	0.0003 (9)
C2	0.0844 (18)	0.0406 (13)	0.0435 (13)	0.0036 (12)	0.0093 (12)	0.0116 (10)
C15	0.0818 (19)	0.0533 (16)	0.0810 (19)	0.0250 (14)	0.0273 (15)	0.0276 (14)
C10	0.0566 (13)	0.0499 (14)	0.0410 (12)	0.0122 (11)	0.0090 (10)	0.0039 (10)
C11	0.0554 (13)	0.0608 (15)	0.0465 (13)	0.0065 (12)	0.0171 (11)	−0.0070 (11)
C3	0.0767 (17)	0.0511 (14)	0.0449 (13)	−0.0041 (12)	0.0228 (12)	0.0099 (11)
C7	0.0530 (13)	0.0463 (13)	0.0350 (11)	0.0105 (10)	0.0079 (9)	0.0040 (9)
C1	0.0678 (16)	0.0462 (14)	0.0581 (15)	0.0152 (12)	0.0103 (13)	0.0121 (11)

### Geometric parameters (Å, °)

**Table:**

Cl2—C6	1.722 (2)	C8—C6	1.384 (3)
Cl1—C7	1.722 (2)	C13—C12	1.384 (3)
O1—C8	1.367 (2)	C13—C14	1.386 (3)
O1—C9	1.394 (2)	C13—H13	0.9300
N1—C7	1.320 (3)	C12—C11	1.371 (3)
N1—C5	1.335 (3)	C12—H12	0.9300
O2—C14	1.358 (3)	C2—C3	1.364 (4)
O2—C15	1.426 (3)	C2—C1	1.368 (4)
N3—C6	1.322 (3)	C2—H2	0.9300
N3—C5	1.330 (3)	C15—H15A	0.9600
N2—C4	1.327 (3)	C15—H15B	0.9600
N2—C3	1.337 (3)	C15—H15C	0.9600
C4—N4	1.328 (3)	C10—C11	1.386 (3)
C4—C5	1.498 (3)	C10—H10	0.9300
N4—C1	1.338 (3)	C11—H11	0.9300
C9—C10	1.370 (3)	C3—H3	0.9300
C9—C14	1.393 (3)	C1—H1	0.9300
C8—C7	1.380 (3)		
			
C8—O1—C9	118.03 (16)	N3—C6—C8	123.3 (2)
C7—N1—C5	115.60 (19)	N3—C6—Cl2	117.29 (17)
C14—O2—C15	117.23 (18)	C8—C6—Cl2	119.36 (17)
C6—N3—C5	116.07 (19)	C3—C2—C1	117.1 (2)
C4—N2—C3	115.4 (2)	C3—C2—H2	121.4
N2—C4—N4	127.39 (19)	C1—C2—H2	121.4
N2—C4—C5	116.39 (19)	O2—C15—H15A	109.5
N4—C4—C5	116.22 (19)	O2—C15—H15B	109.5
N3—C5—N1	126.23 (19)	H15A—C15—H15B	109.5
N3—C5—C4	117.52 (18)	O2—C15—H15C	109.5
N1—C5—C4	116.23 (19)	H15A—C15—H15C	109.5
C4—N4—C1	115.1 (2)	H15B—C15—H15C	109.5
C10—C9—C14	121.26 (19)	C9—C10—C11	119.7 (2)
C10—C9—O1	123.52 (18)	C9—C10—H10	120.1
C14—C9—O1	115.15 (18)	C11—C10—H10	120.1
O1—C8—C7	122.9 (2)	C12—C11—C10	119.8 (2)
O1—C8—C6	122.0 (2)	C12—C11—H11	120.1
C7—C8—C6	114.86 (19)	C10—C11—H11	120.1
C12—C13—C14	120.3 (2)	N2—C3—C2	122.3 (2)
C12—C13—H13	119.9	N2—C3—H3	118.8
C14—C13—H13	119.9	C2—C3—H3	118.8
O2—C14—C13	125.57 (19)	N1—C7—C8	123.9 (2)
O2—C14—C9	116.05 (18)	N1—C7—Cl1	116.80 (18)
C13—C14—C9	118.4 (2)	C8—C7—Cl1	119.30 (16)
C11—C12—C13	120.6 (2)	N4—C1—C2	122.6 (2)
C11—C12—H12	119.7	N4—C1—H1	118.7
C13—C12—H12	119.7	C2—C1—H1	118.7

### Hydrogen-bond geometry (Å, °)

**Table:**

Cg is the centroid of the C9–C14 ring.

**Table:**

*D*—H···*A*	*D*—H	H···*A*	*D*···*A*	*D*—H···*A*
C1—H1···Cg^i^	0.93	2.87	3.760 (3)	161

Symmetry codes: (i) *x*+1/2, −*y*−1/2, *z*+1/2.
